# Flavomycin restores colistin susceptibility in multidrug-resistant Gram-negative bacteria

**DOI:** 10.1128/msystems.00109-24

**Published:** 2024-05-02

**Authors:** Ying Huang, Yan Zhu, Hui-Ying Yue, Yi-Yun Liu, Li-Min Deng, Luchao Lv, Chengzhen Wang, Jun Yang, Jian-Hua Liu

**Affiliations:** 1 State Key Laboratory for Animal Disease Control and Prevention, Guangdong Laboratory for Lingnan Modern Agriculture, College of Veterinary Medicine, South China Agricultural University, Guangzhou, Guangdong, China; 2 Key Laboratory of Zoonosis of Ministry of Agricultural and Rural Affairs, Guangdong Provincial Key Laboratory of Veterinary Pharmaceutics Development and Safety Evaluation, Guangzhou, Guangdong, China; 3 Systems Biology Center, Tianjin Institute of Industrial Biotechnology, Chinese Academy of Sciences, Tianjin, China; 4 Guangdong Provincial Key Laboratory of Pharmaceutical Bioactive Substances, School of Basic Medical Sciences, Guangdong Pharmaceutical University, Guangzhou, China; Hangzhou Institute for Advanced Study, UCAS, Hangzhou, Zhejiang, China

**Keywords:** *Enterobacterales*, flavomycin, colistin, MDR, synergistic mechanism

## Abstract

Polymyxin is used as a last resort antibiotics for infections caused by multi-drug resistant (MDR) Gram-negative bacteria and is often combined with other antibiotics to improve clinical effectiveness. However, the synergism of colistin and other antibiotics remains obscure. Here, we revealed a notable synergy between colistin and flavomycin, which was traditionally used as an animal growth promoter and has limited activity against Gram-negative bacteria, using checkerboard assay and time–kill curve analyses. The importance of membrane penetration induced by colistin was assessed by examining the intracellular accumulation of flavomycin and its antimicrobial impact on *Escherichia coli* (*E. coli*) strains with truncated lipopolysaccharides. Besides, a mutation in the flavomycin binding site was created to confirm its role in the observed synergy. This synergy is manifested as an augmented penetration of the *E. coli* outer membrane by colistin, leading to increased intracellular accumulation of flavomycin and enhanced cell killing thereafter. The observed synergy was dependent on the antimicrobial activity of flavomycin, as mutation of its binding site abolished the synergy. *In vivo* studies confirmed the efficacy of colistin combined with flavomycin against MDR *E. coli* infections. This study is the first to demonstrate the synergistic effect between colistin and flavomycin, shedding light on their respective roles in this synergism. Therefore, we propose flavomycin as an adjuvant to enhance the potency of colistin against MDR Gram-negative bacteria.

**IMPORTANCE:**

Colistin is a critical antibiotic in combating multi-drug resistant Gram-negative bacteria, but the emergence of mobilized colistin resistance (mcr) undermines its effectiveness. Previous studies have found that colistin can synergy with various drugs; however, its exact mechanisms with hydrophobic drugs are still unrevealed. Generally, the membrane destruction of colistin is thought to be the essential trigger for its interactions with its partner drugs. Here, we use clustered regularly interspaced palindromic repeats (CRISPR)–CRISPR-associated protein 9 (Cas9) for specifically mutating the binding site of one hydrophobic drug (flavomycin) and show that antimicrobial activity of flavomycin is critical for the synergy. Our results first give the evidence that the synergy is set off by colistin's membrane destruction and operated the final antimicrobial function by its partner drugs.

## INTRODUCTION

The rise of antimicrobial resistance poses a significant threat to global health, while this issue is magnified by a declining drug discovery rate, leading to fewer effective treatments (1). Multi-drug resistant (MDR) Gram-negative bacteria is a major health challenge. The World Health Organization has classified extended-spectrum β-lactamase (ESBL)-producing and carbapenemase-resistant *Enterobacterales* (CRE) species as high priority threats. Colistin, a drug from the 1960s, remains one of few effective treatments against these bacteria (2); it has been revived as the last-resort antibiotic to treat severe infections caused by ESBL-producing *Enterobacterales* and CRE. The bactericidal mechanism of colistin is mainly involved in initial electrostatic interaction with the negatively charged lipid A phosphate components of lipopolysaccharides (LPS) and subsequent disorganization of the outer membrane (OM) (3). However, the plasmid-borne colistin resistance gene *mcr-1* hinders the interaction between colistin and OM by modifying the LPS lipid A phosphate components (4, 5). The recent global spread of this gene poses a significant challenge to the effectiveness of colistin and has garnered substantial attention worldwide (6–9).

Unfortunately, the clinically used polymyxin antibiotics usually exhibit dose-limiting nephrotoxicity (10). Moreover, it has been observed that higher polymyxin B exposures could increase resistant sub-populations in pathogenic bacteria, such as *Acinetobacter baumannii* (11). These highlight the need to combine polymyxin drugs with other antibiotics to combat multi-drug resistant Gram-negative bacteria. So far, numerous studies have revealed that colistin exhibits a synergy with some drugs, encompassing both antibiotics and non-antibiotic drugs (12). Interestingly, colistin also demonstrates synergism with many drugs targeting Gram-positive bacteria, such as rifampicin, novobiocin, clarithromycin, erythromycin, and fosfomycin (13). Some researchers hypothesized that this synergistic mechanism might stem from membrane disruption, which facilitates the partner drugs to enter into bacteria to exert their antimicrobial activity (14). This opinion is indirectly supported by a study in which the synergy of colistin and rifampicin combination disappeared when mutants resistant to both drugs are induced (13). However, direct evidence corroborating that partner drugs, like rifampicin, can penetrate and perform antimicrobial activities, facilitated by colistin’s membrane disruption, remains elusive.

As plasmid-mediated horizontal transfer is central to the dissemination of resistance, inhibiting plasmid conjugation is an alternative strategy to combat MDR (15). Previous studies showed that flavomycin, an antimicrobial agent derived from *Streptomyces* spp., has an inhibitory effect on conjugation of resistance plasmids (16, 17). It was primarily employed as a growth promoter in livestock animals, with good antimicrobial activity against Gram-positive pathogens by targeting the peptidoglycan glycosyltransferase (PGT) domain of class A penicillin-binding protein (PBP) and mono-functional PGT (18). Due to the hydrophilic network formed by LPS in the OM of Gram-negative bacteria, the hydrophobic nature of flavomycin hampers its penetration and results in its limited efficacy against Gram-negative bacteria (19).

While studying the inhibition of flavomycin on conjugation, we unexpectedly noted a remarkably reduced conjugation frequency in the presence of flavomycin and colistin. Through checkerboard assay and time–kill curve analysis, we validated the synergistic antibacterial effect between flavomycin and colistin. Furthermore, we investigated the synergistic bactericidal mechanism between colistin and flavomycin and assessed their therapeutic potential *in vivo*.

## RESULTS

### Colistin induced overestimation of flavomycin inhibition on plasmid conjugation

In alignment with previous reports (16, 17), flavomycin inhibits plasmid conjugation in a dose-dependent manner (Table S1; Fig. S1). Interestingly, the inhibition of flavomycin on the conjugation of *mcr-1-*carrying plasmids was much greater than that on *bla*_NDM_- or *bla*_CTX-M_-carrying plasmids (*P* < 0.05) (Fig. S1; Table S1), specifically, an IncHI2 plasmid SHP26 harboring *mcr-1* gene and *bla*_CTX-M_ genes. When colistin or cephalosporin was used for selecting transconjugants of this plasmid, a remarkable disparity was observed. The number of transconjugants on colistin agar medium was significantly lower than those on cefotaxime agar medium (Fig. S1), suggesting that the selection pressure of antibiotics could bias the assessment of flavomycin activity on plasmid conjugation.

**Fig 1:**
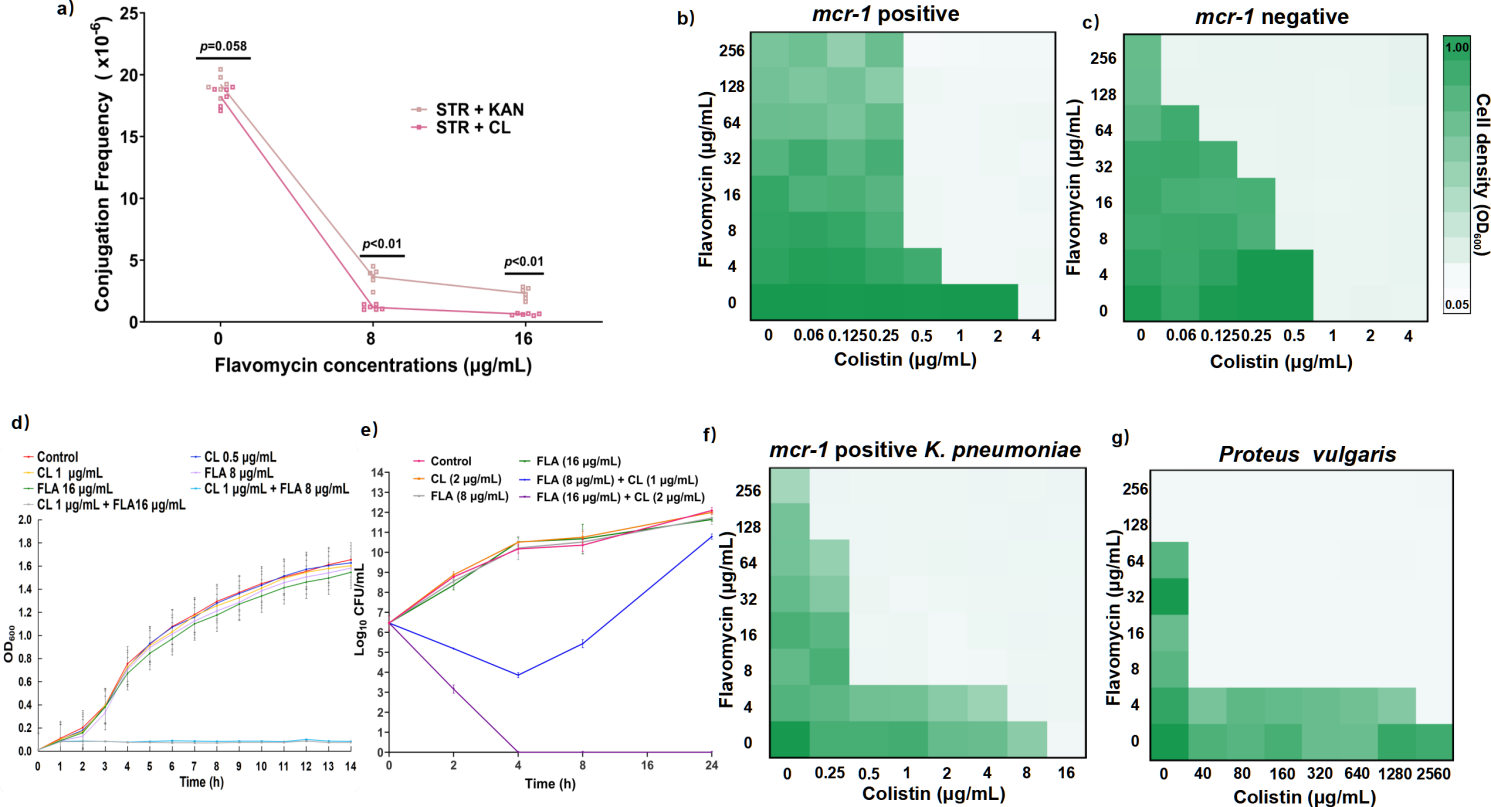
The effects of flavomycin on conjugation and its interaction with colistin. (**a)** Flavomycin demonstrates a more profound dose-dependent inhibition of conjugation frequencies in *E. coli* strains (BW25113/pHNSHP45-*kan*) in the presence of colistin. Lines are painted in various colors according to different selective plates. The selective plate with streptomycin and colistin was in pale pink, and the plate with streptomycin and colistin was in pink. Data are done in three duplicates. Checkerboard broth micro-dilution assays between flavomycin and colistin in *mcr-1*-positive (**b**) or *mcr-1*-negative (**c**) *E. coli*. (**d)** Growth curve of *E. coli* after treatment with different combinations of flavomycin and colistin. FLA, flavomycin; CL, colistin. (**e)** Time-dependent killing of *E. coli* treated with flavomycin combined with colistin. Data are representative of three independent experiments, mean ± SD. Checkerboard broth microdilution assays between flavomycin and colistin in *Klebsiella pneumoniae* (**f**) or *Proteus vulgaris* (**g**). In the heat map, the darker the color, the greater the bacteria density. Data represent the mean absorbance of two biological replicates.

To confirm the influence of antibiotic selection on conjugation inhibition, we introduced a kanamycin-resistant gene into an *mcr-1*-positive *Escherichia coli* plasmid (pHNSHP45-*kan*). The conjugation frequencies of this plasmid were detected using the selection plates containing streptomycin with the addition of colistin or kanamycin (Fig. 1a; Table S1). As anticipated, the plasmid conjugation frequency detected with colistin selection was markedly lower than that with kanamycin selection, suggesting that colistin selection may overestimate the inhibitory effect of flavomycin on conjugation.

### Synergistic antibacterial activity of flavomycin with colistin

We hypothesized that the enhanced conjugation inhibition of flavomycin by colistin potentially stems from their synergistic antibacterial killing. The results showed that flavomycin reduced minimum inhibitory concentration (MIC) of colistin-susceptible isolates from 0.5–1 to 0.0625–0.25 µg mL^−1^ (Table S2; Fig. 1c), with a fractional inhibitory concentration index (FICI) of 0.19–0.49 (synergistic). In addition, flavomycin and colistin also demonstrated synergy against colistin-resistant bacteria harboring *mcr-1* or resistance-conferring chromosomal mutations, characterized by FICI ranging from 0.023 to 0.375 (synergistic). Notably, flavomycin (2–128 µg mL^−1^) restored colistin susceptibility, reducing the colistin MIC from 4–1,024 to 0.25–16 µg mL^−1^ (Table S2; Fig. 1b). The growth curve presented that sub-lethal levels of either flavomycin (8–16 µg mL^−1^) or colistin (1 µg mL^−1^) alone only slightly affect the growth of colistin-resistant *E. coli*; however, their combinations (1 µg mL^−1^ colistin + 8 or 16 µg mL^−1^ flavomycin) achieved a complete growth inhibition (Fig. 1d). The time–kill curve showed that flavomycin (16 µg mL^−1^) in combination with colistin (2 µg mL^−1^) effectively eradicated *E. coli* within 4 h (Fig. 1e). In addition, the combination therapy effectively killed colistin-resistant Gram-negative species, including *Proteus vulgaris* and *mcr-1* carrying *Klebsiella pneumoniae* (Table S2; Fig. 1f and g).

### Colistin augmented flavomycin activity by enhancing its penetration through bacterial OM

**Fig 2:**
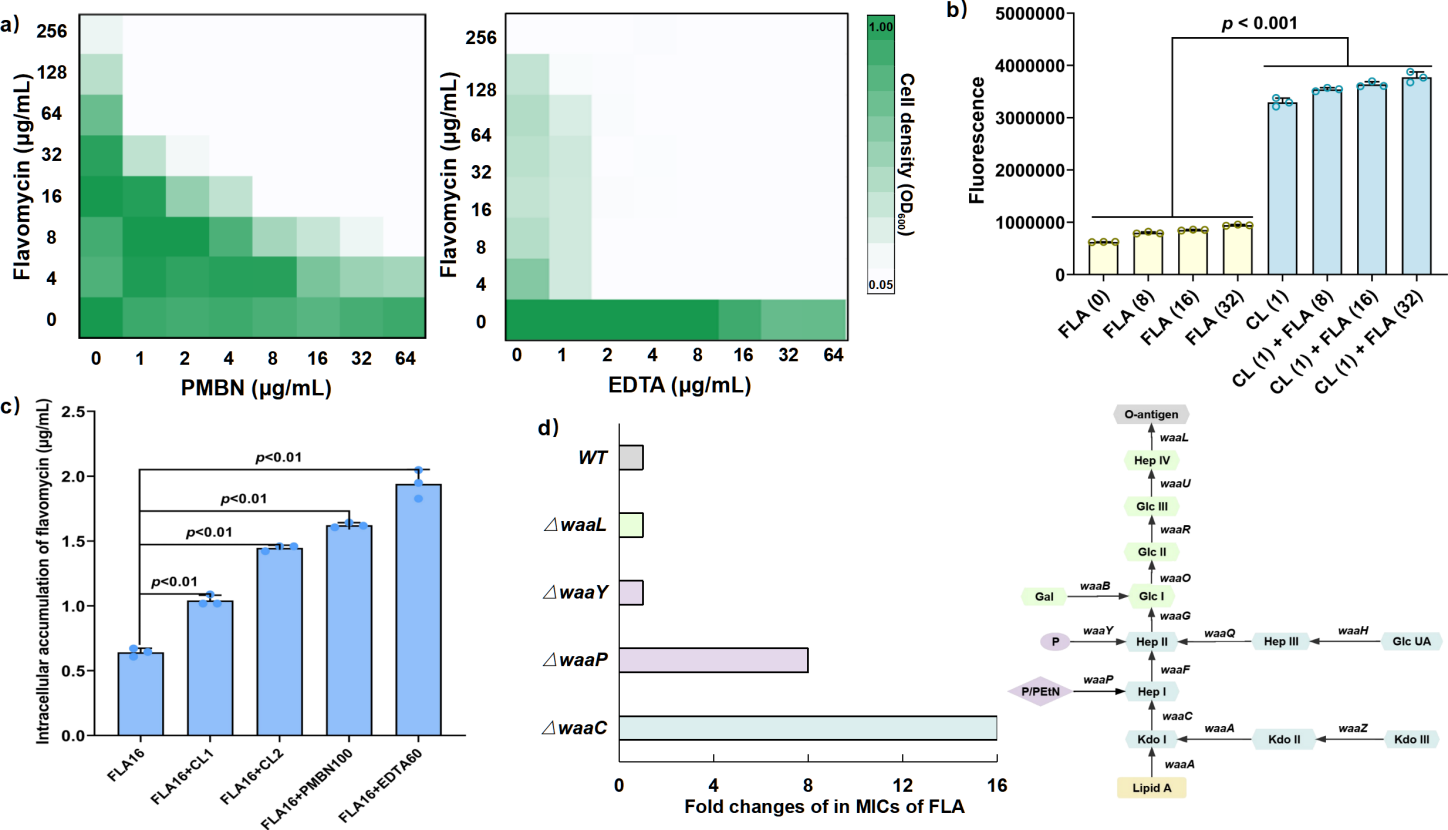
Mechanism of flavomycin in combination with colistin against bacteria (**a)** Checkerboard broth microdilution assays between flavomycin and PMBN or EDTA in *E. coli* at 37°C. Dark regions represent higher cell density. Checkerboard data are representative of two biological replicates. (**b)** Increased permeability of the OM of *E. coli* treated with drug combination. Permeability was evaluated by measuring the fluorescence intensity of 1-*N*-phenylnaphthylamine (NPN) after exposure to either increasing concentrations of flavomycin, colistin, or colistin plus flavomycin. (**c)** Intracellular flavomycin levels were quantified using liquid chromatography with tandem mass spectrometry. This analysis compared flavomycin accumulation when administered alone versus in combination with colistin, PMBN, and EDTA. (**d)** Fold change in the MIC of flavomycin against *E. coli* with various truncations in the core oligosaccharide (OS) (right). Light green represents the outer-core OS; light blue represents inner-core OS; purple represents the core OS phosphates (left).

The OM of Gram-negative bacteria forms a hydrophobic barrier that renders many anti-Gram-positive antibiotics, including flavomycin, ineffective. Since colistin initially targets the Gram-negative OM, we explored whether the potentiation of colistin with flavomycin is related to the OM disruption of colistin. Experiments combining flavomycin with OM-targeting polymyxin B nonapeptide (PMBN) or EDTA revealed significant synergy (FICI = 0.0234–0.1875 or 0.0256, respectively) (Fig. 2a). Besides, OM was significantly damaged by colistin alone or in combination with flavomycin; however, flavomycin alone did not show such effect (Fig. 2b). We also observed enhanced intracellular accumulation of flavomycin in the presence of colistin, PMBN, or EDTA (Fig. 2c; Fig. S4), suggesting that compromising OM could facilitate intracellular accumulation of flavomycin.

On the other hand, the integrity of the OM is maintained through the ion bridges between LPS molecules and divalent ions (e.g., Mg^2+^, Ca^2+^). The addition of an excess amount of divalent cations has the capacity to amplify this interaction, which, in turn, inhibits the binding of colistin to LPS (20). We found that exogenous supplementation with Mg^2+^ when beyond 15 mM abrogated the synergy of flavomycin and colistin (Fig. S2a), corroborating our hypothesis that the synergy is primarily from OM disorganization caused by colistin. To further investigate the role of LPS on flavomycin activity, we assessed its efficacy against *E. coli* mutants expressing truncated LPS core oligosaccharide (OS) variants. Deeper truncation (BW25113∆*waaC::kan*) or removal of core OS phosphate residues (BW25113∆*waaP::kan*) significantly reduced flavomycin MIC (Fig. 2d; Table S3a). This finding indicated that the disruption of LPS could increase the sensitivity of *E. coli* to flavomycin.

### Mutating the binding site of flavomycin abolished the synergy of colistin and flavomycin

**Fig 3:**
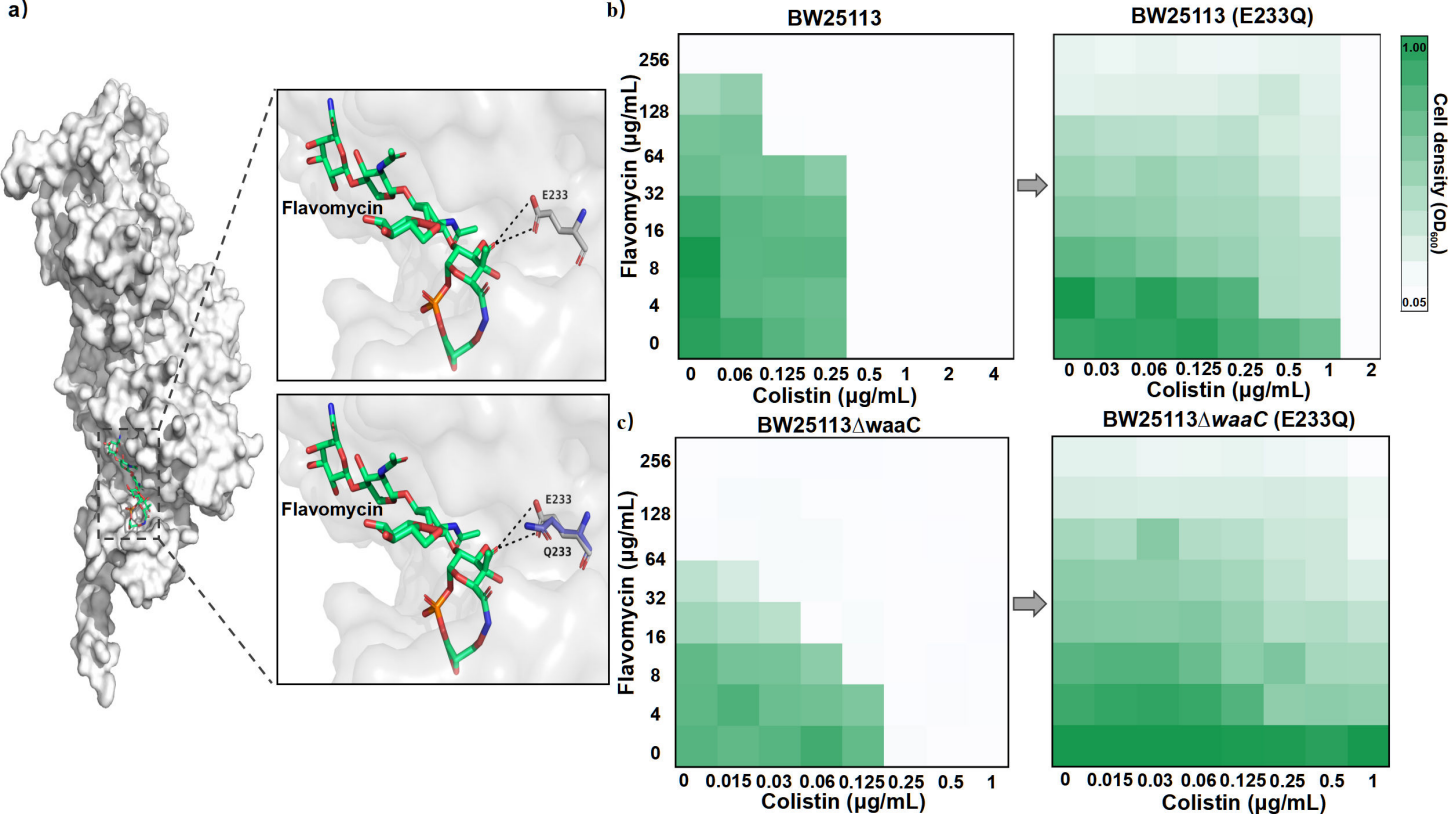
Colistin potentiates flavomycin against *E. coli.* (**a)** The model of *E. coli* PBP 1B binding with flavomycin. The flavomycin chemical structure with its binding site on peptidoglycan glycosyltransferase (PGT) (donor site) shown in surface mode with the ligand drawn in stick. The effect of PBP 1B (E233Q) on the FICI for wild-type *E. coli* BW25113 (**b**) and *E. coli* BW25113∆*waaC* (**c**). Data are representative of three independent experiments, and the mean of three biological replicates is shown. Error bars represent the SD, and *P* values were determined using an unpaired, two-tailed Student’s *t*-test.

**Fig 4:**
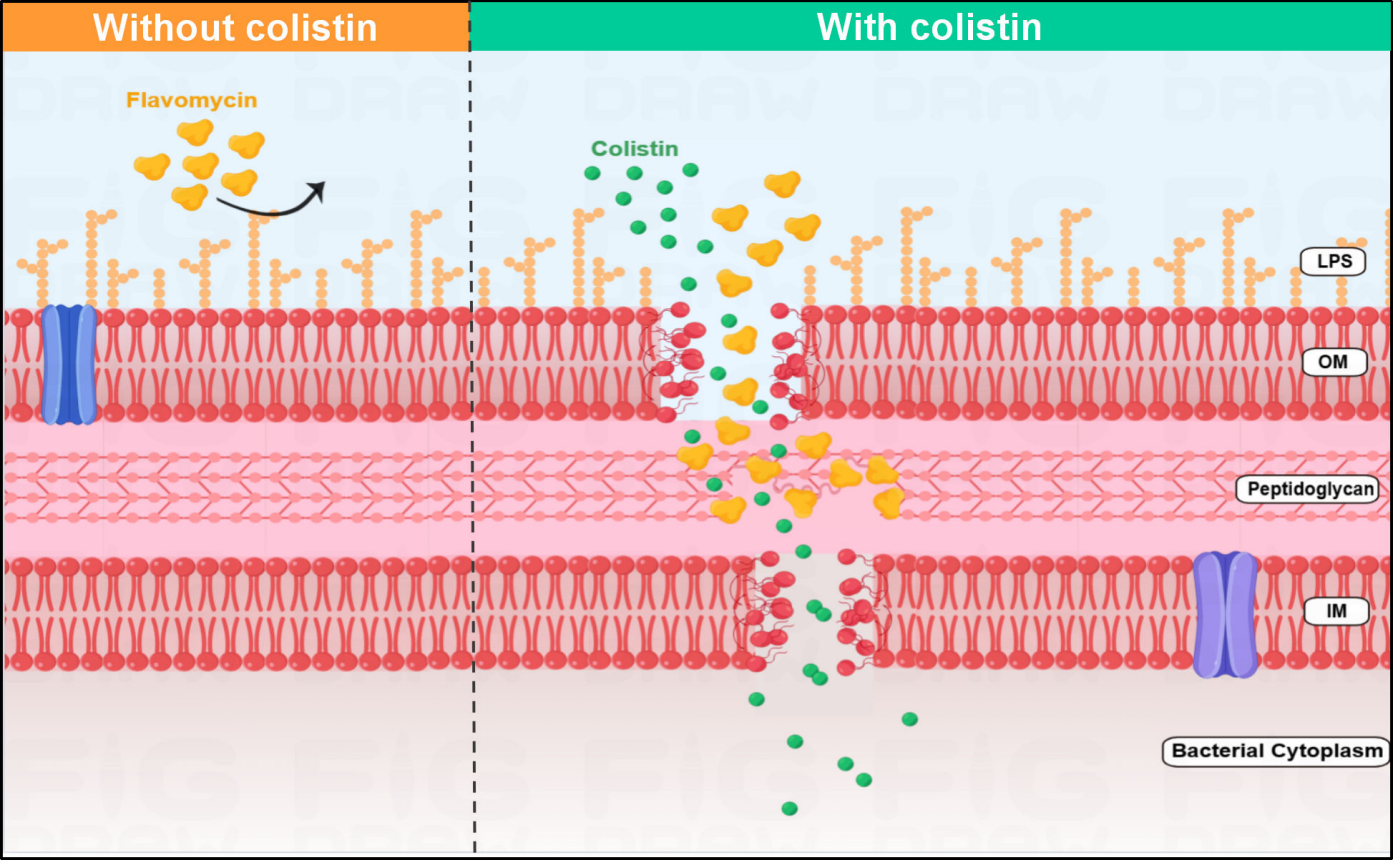
Scheme of the synergy of flavomycin in combination with colistin. Colistin enhances the susceptibility of *E. coli* to flavomycin through membrane-mediated mechanisms by interacting with LPS in the OM. These interactions prompt the disruption of the OM, consequently leading to the intracellular accumulation of flavomycin, which enables flavomycin to exert its antimicrobial function by targeting the periplasm.

We further examined the role of flavomycin in the synergism using a strain with mutated flavomycin target. Previous studies suggest that flavomycin targets the PGT domain of class A PBP and monofunctional PGT characterized by five highly conserved amino acid motifs: EDxxFxxHxG, GxSTxTQQ, RKxxE, KxxIxxxYxN, and RxxxL (21). These motifs are vital to the formation of an active site, and mutations in these motifs can reduce the binding affinity of flavomycin, leading to increased bacterial resistance (22). We introduced a mutation E233Q (one of these essential amino acid residue sites, Fig. 3a; Fig. S3) to the PBP 1B in LPS-deficient *E. coli* BW25113∆*waaC*, using the CRISPR–Cas9 technique (23). This strain was selected as the LPS truncation amplifies its susceptibility to flavomycin, evident by a 16-fold reduction of its MIC (Fig. 2d; Fig. S3). The result revealed that the mutation E233Q made BW25113∆*waaC* resistant to flavomycin, as the MIC increased from 16 to 256 μg mL^−1^ (Table S3). The E233Q mutation in PBP 1B resulted in the FICI changing from ≤0.5 (synergistic) to 1-2 (indifferent) for BW25113 and BW25113*∆waaC* (Table S2; Fig. 3). Besides, neither the *waaC* deletion nor the E233Q mutation in *E. coli* BW25113 impacted bacterial growth (Fig. S2b). Our results suggest that colistin-induced disruption of the OM enabled flavomycin to inhibit PGT function, leading to bacterial death (Fig. 4).

### The synergistic efﬁcacy of flavomycin and colistin *in vivo*

**Fig 5:**
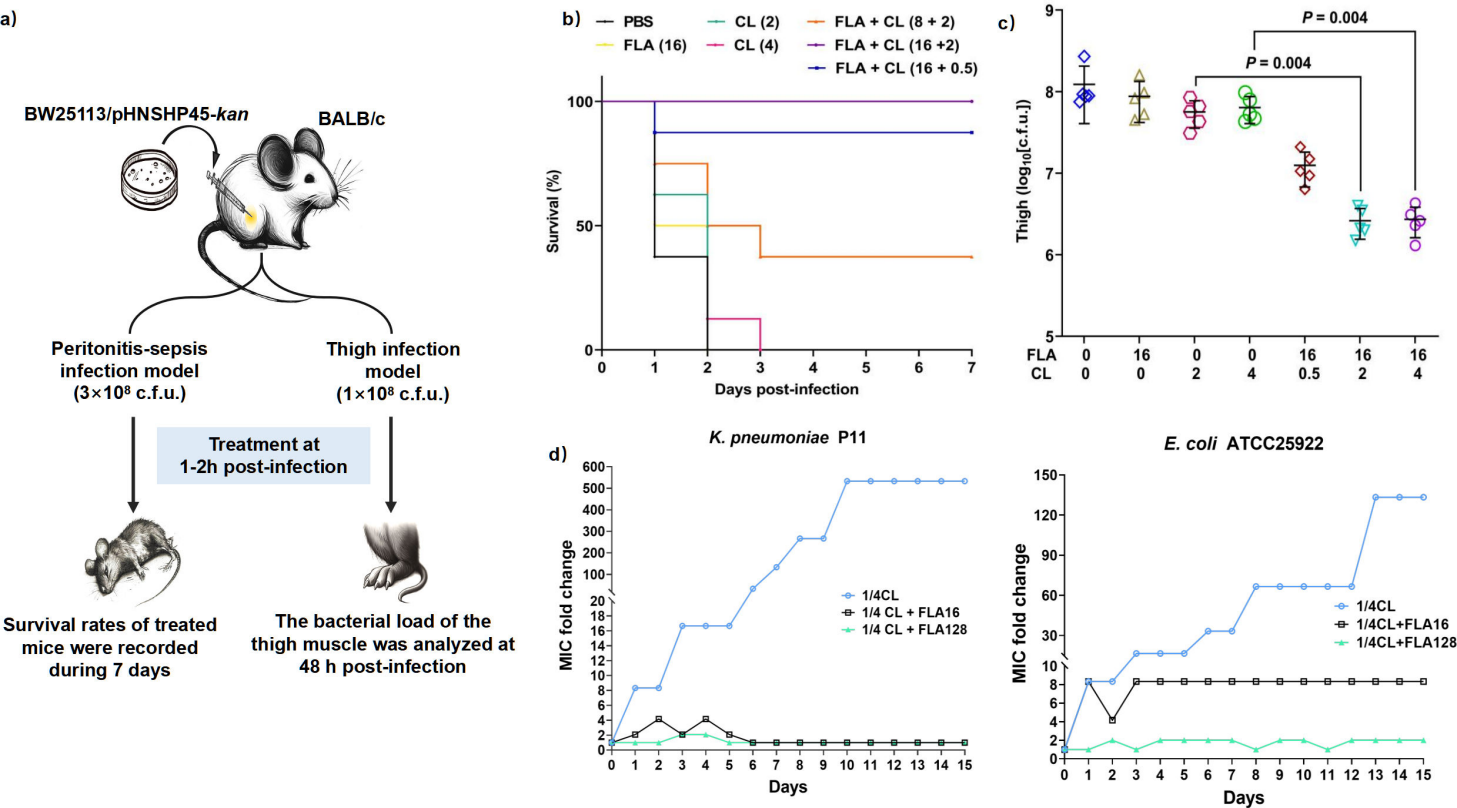
Efﬁcacy of flavomycin combined with colistin in a mouse infection model. (**a)** Scheme of the experimental protocol for the mouse peritonitis–sepsis model and mouse thigh infection model. (**b)** Survival rates of mice in the mouse peritonitis–sepsis model (*n*  =  8). Increased survival rates of mice treated with a combination of flavomycin and colistin over 7 days. (**c)** Bacterial load in the mouse thigh infection model (*n*  =  5). The bacterial load of the right thigh muscle infected with a non-lethal dose of *E. coli* (1.0  ×  10^8^ CFU/mL) decreased significantly after a single intraperitoneal dose of colistin (2 mg kg^−1^) plus flavomycin (16 mg kg^−1^). *P* values were determined using a two-sided, Mann–Whitney *U*-test. (**d**) Flavomycin suppresses the evolution of colistin resistance. Serial passages of *E. coli* ATCC25922 at sub-inhibitory concentrations of colistin (1/4 MIC) or combination with flavomycin (16 or 128 µg mL^−1^). The MIC values were measured every 24 h. Data are representative of three independent experiments, and the mean of three biological replicates is shown.

To explore the *in vivo* efficiency of this combination, we conducted two mouse infectious models (Fig. 5a). As a result, neither colistin nor flavomycin alone was effective in circumventing a lethal infection caused by *mcr-1*-positive *E. coli* (BW25113/pHNSHP45-kan). However, the combination of 16 mg kg^−1^ of flavomycin and 2 mg kg^−1^ of colistin provides full protection against the infection for 7 days post infection (Fig. 5b). Additionally, the combination significantly reduced the bacterial load in mouse thigh muscle (Fig. 5c). Therefore, the combination of flavomycin and colistin might represent a promising approach to address the MDR Gram-negative bacterial infections.

### Flavomycin restricts the evolution of colistin resistance

To gain a better understanding of the effect of flavomycin on the development of colistin resistance, we performed serial passages of *E. coli* or *K. pneumoniae* with colistin (0.25 × MIC) in the presence or absence of sub-MIC (16 or 128 µg mL^−1^) of flavomycin for 15 days. Sequential passaging with colistin alone for 15 days led to emergence of resistance, as shown by a marked increase in MIC: *E. coli* from 0.03 to 4 µg mL^−1^ (133-fold), and *K. pneumoniae* from 0.25 to 128 µg mL^−1^ (512-fold). However, combining flavomycin with colistin decelerated this resistance evolution (Fig. 5d and e). This result indicated that flavomycin could restrict the emergence of colistin resistance in bacteria.

## DISCUSSION

The rapid spread of MDR Gram-negative bacteria has curtailed effective antimicrobial options, leading to the revival of several “old drugs” serving as last-resort antibiotics (3). Combination therapies are suggested to maintain antibiotic clinical efficacy against Gram-negative bacterial infections. Colistin shows synergy with a range of drugs, such as flavonoids, rifampicin, tigecycline, macrolides, glycopeptides, and equisetin (12, 24). Notably, some anti-Gram-positive agents, including vancomycin, daptomycin, and linezolid, have shown synergy with colistin in killing colistin-resistant Gram-negative bacteria (25). These antibiotics exhibit distinct bactericidal mechanisms, but primarily target intracellular components, such as cell wall synthesis (vancomycin), transcription process (daptomycin), or protein synthesis (linezolid) (25). However, the mechanism of synergy between colistin and these drugs remains unclear.

As colistin exerts antibacterial activity by binding to LPS in Gram-negative bacterial OM, destabilizing the membrane and eventually leading to cell death (5), the synergy between colistin and hydrophobic drugs first may rely on colistin-induced OM damage (13). However, given that hydrophobic antibiotics are typically excluded by the intact OM, and *mcr-1*-expressing strains resist colistin, it raises questions: does colistin maintain its membrane-disrupting activity against resistant strains, and does its synergy with hydrophobic drugs still depends on this feature? What is the principal driver of this synergy? A study by MacNair et al. (13) offers a hypothesis on this matter, which revealed the changing synergy between colistin and rifampicin in the presence of induced resistance to both drugs. They suggest that *mcr-1* impedes the role of the fatty-acyl tail in self-promoted uptake and lysis, but retains its importance in OM disruption (13). However, it remains unclear whether colistin or the partnered antibiotic primarily contributes to the antimicrobial activity. Our unexpected finding of colistin’s synergy with flavomycin against colistin-resistant strains suggested a potential parallel to MacNair’s hypothesis. Here, we elucidated the mechanism underlying the synergy between flavomycin and colistin.

Given the mutual exclusion between flavomycin and the OM, and the observed increase in OM permeability and intracellular accumulation of flavomycin facilitated by colistin (Fig. 2d; Fig. S4), we hypothesized that colistin-induced OM damage might underpin the synergy between flavomycin and colistin. To delve deeper, we truncated the LPS of *E. coli*, a major obstacle to flavomycin. As expected, LPS truncation enhanced flavomycin’s bactericidal efficacy against *E. coli*. Interestingly, the synergy between colistin and flavomycin ceased upon inactivation of the flavomycin target (Table S2; Fig. 3). These results confirmed our speculation and further explained the synergistic mechanism of colistin and flavomycin that colistin-induced damage allowed the entry of flavomycin to exert antimicrobial effects. Besides, *in vivo* tests showed that this combination effectively combated resistant strains. The identification of the synergistic interaction between flavomycin and colistin is highly significant. It aids in extending flavomycin’s clinical applications and helps in diminishing the health risks posed by multidrug-resistant Gram-negative bacteria.

While these findings are promising, further study is warranted to understand the potential benefits and risks of this combination. For instance, in our study, we noted that colistin and flavomycin, when synergistic, could effectively bypass the resistance evolution of either drug. However, the precise relationship between their synergistic effects and the suppression of antibiotic-resistant evolution remains elusive due to the involvement of various factors. This phenomenon, commonly reported in similar studies on drug synergy, also lacks comprehensive explanation. Consequently, further research is required to unravel the connection between such synergistic interactions and their role in diminishing antibiotic resistance evolution. Besides, *in vivo* studies and clinical trials would be required to evaluate the safety and effectiveness of this drug combination in patients. Additionally, potential mechanisms of resistance to this combined treatment would need to be investigated to ensure its long-term efficacy. Furthermore, flavomycin’s structure, which includes a phosphoglycolipid moiety with a unique C25 isoprenoid side chain, a heptasaccharide (seven sugar units), and a phosphoric acid group , results in an extended fatty chain (19). This characteristic gives flavomycin an unusually long half-life in mammals, which negatively impacts its pharmacokinetics and bioavailability, thus limiting its systemic use in humans (19). Thus, optimizing flavomycin to enhance its clinical applications is of vital importance.

Moreover, given the OM of Gram-negative bacteria, which acts as a primary defense against numerous antibiotic classes, we propose that drugs similar to colistin, such as pentamidine (26), which enhance OM damage, could be instrumental in facilitating the penetration of antibiotics typically effective only against Gram-positive bacteria. Therefore, the identification or creation of additional antimicrobial drugs targeting the OM, alongside their synergy with existing hydrophobic drugs, may provide another strategy to combat the critical issue of drug resistance. Furthermore, our research also suggests that it may be worthwhile to investigate whether the inhibitor has a synergy with drugs used for screening conjugants.

Collectively, our findings corroborate prior hypotheses that the synergy between colistin and hydrophobic drugs arises from colistin’s disruption of the hydrophobic OM in Gram-negative bacteria. This disruption paves the way for the antibiotic partner to penetrate the cell, thereby manifesting its bactericidal effect. Overall, flavomycin was revived as a promising antibiotic adjuvant, with the capacity to enhance the effectiveness of existing antibiotics against Gram-negative bacteria.

## MATERIALS AND METHODS

### Bacterial strains

The colistin-susceptible or -resistant strains used in this study were collected or obtained in our previous studies (Table S2) (27, 28). The constructed strain (BW25113/pHNSHP45*-kan*) and *E. coli* variants exhibiting different lengths of truncated LPS were created using the λRed recombination system (26, 29).

### Antimicrobial susceptibility tests

Antimicrobial susceptibility tests were conducted for streptomycin, colistin, cefotaxime, and flavomycin using the standard broth micro-dilution method, following the Clinical and Laboratory Standards Institute guidelines (30).

### Bacterial growth assay

Growth kinetics in lysogeny broth (LB) were examined for various concentrations of flavomycin and colistin, either alone or combined, compared to a non-treatment control. Bacteria were grown at 37°C with shaking, starting at an optical density at 600 nm (OD_600_) of 0.01. OD_600_ readings were taken hourly for 14 h.

### Conjugation experiment

Conjugation was conducted using previous methods with modifications (17). *E. coli* C600 derivative with a high-level resistance to streptomycin was used as the recipient strain.

### Checkerboard assays

Synergistic activity of flavomycin and colistin was evaluated by checkerboard assays. In a 96-well microplate, flavomycin was diluted vertically, while colistin was diluted horizontally using Muller–Hinton broth (MHB). Bacterial suspensions were then diluted to 1.5 × 10^6^ CFU/mL in MHB, and 100 µL was added to each well. After incubation for 20 h at 37°C, the FIC index (FICI) was calculated (31): FIC index = MIC_AB_/MIC_A_ + MIC_BA_/MIC_B_ = FIC_A_ + FI_CB_. MIC_A_ is the MIC of compound A alone, MIC_AB_ is the MIC of compound A in combination with compound B, MIC_B_ is the MIC of compound B alone, MIC_BA_ is the MIC of compound B in combination with compound A; FIC_A_ is the FIC of compound A and FIC_B_ is the FIC of compound B. The FIC index cutoff defining synergy was set to ≤0.5.

### Time–kill assay

Overnight culture of BW25113/pHNSHP45 at a 1:100 ratio was inoculated into fresh LB broth for 4 h. The bacteria were collected by centrifugation, and cells were then resuspended in MHB at 10^6^–10^7^ CFU/mL. The bacteria were treated with flavomycin, colistin, or both drugs, respectively. Subsequently, 10-fold serially diluted suspensions were plated on MHA plates and incubated overnight at 37°C. Bacterial colonies were counted, and the primary CFU/mL was calculated. All experiments were performed with at least three biological replicates.

### OM disruption assay

Fluorescent probe 1-*N*-phenylnaphthylamine (NPN) (10 µM) was used to evaluate the OM integrity of *E. coli* following treatments, with the excitation wavelength at 350 nm and emission wavelength at 420 nm.

### Construction of the mutant with a point mutation in the target of flavomycin

A point mutation at the amino acid residue E233, which was essential for the bactericidal effect of flavomycin, was introduced by the CRISPR–Cas9 system (18). Two sgRNAs were designed on either side of the coding region of E233 amino acid residue, and the template for repair contains a mutation (G–C) at the coding region of E233 (23).

### Intracellular antibiotic accumulation

*E. coli* single colonies from LB agar plates were inoculated into the LB broth and incubated at 37°C with shaking at 200 rpm until reaching the logarithmic growth phase. The cultures were then diluted 1:100 into 50 mL of fresh sterile LB broth and incubated at 37°C with shaking at 200 rpm until the OD_600_ reached approximately 0.5. The cultures were centrifuged at 4°C and 3,000 × *g* for 10 min. The bacterial pellet was collected and washed three times with phosphate-buffered saline (PBS) (0.01 M, pH 7.4). The bacteria were resuspended in fresh sterile PBS (0.01 M, pH 7.4), aliquoted into sterile 1.5-mL tubes, and adjusted to a concentration of 1,010 CFUs/mL. Flavomycin (16 µg/mL) alone or a combination of flavomycin and colistin was added, mixed, and incubated at 37°C with shaking for 15 min. The cultures were then centrifuged at 4°C at 12,500 rpm for 2 min, and the bacterial pellet was collected. The pellet was resuspended in 400 µL of sterile water and subjected to three freeze–thaw cycles (each 3 min) in liquid nitrogen and a 65°C water bath to lyse the bacteria effectively. The lysates were centrifuged at 12,500 rpm for 2 min, and the supernatant was collected. The remaining bacterial pellet was resuspended in 200 µL of methanol, vortexed, and centrifuged at 12,500 rpm to collect the supernatant. The two supernatants were combined and centrifuged at 12,500 rpm for 10 min, and the supernatant was collected for analysis. The content of flavomycin in the supernatant was quantitatively measured by liquid chromatography with tandem mass spectrometry. The liquid chromatography column was a Shimadzu C18 column (2.1 × 100 mm, 3 µm). The injection volume was 1 µL, with 10 mM ammonium acetate in water as mobile phase A and acetonitrile as mobile phase B. The gradient elution ratio is as follows: 0.0–0.4 min, 95% A; 0.4–0.6 min, 95% A; 0.6–3.0 min, 2% A; 3.0–3.4 min, 2% A; 3.4–10.0 min, 95% A. The injection volume is 7 µL. Multiple reaction monitoring in the negative ion mode was used to quantify intracellular flavomycin, targeting *m*/*z* = 790.0/554.3 (qualifying ion) and *m*/*z* = 790.0/575.9 (quantifying ion).

### Resistance development studies

*E. coli* ATCC 25922 and *K. pneumonia* P11 at exponential phase were diluted (1:1,000) into fresh MHB in the presence of either colistin alone or colistin in combination with flavomycin. Following a 24-h incubation at 37°C, the MIC of the cultures was assessed. The cultures were further diluted with drug-containing fresh MHB for the next passage, and the fold change in colistin MIC was calculated for 15 days.

### Mouse infection model

In mouse peritonitis–sepsis model, mice were intraperitoneally injected with a lethal dose of *E. coli* BW25113. Mice received either PBS, colistin (2 and 4 mg kg^−1^), flavomycin (16 mg kg^−1^), or combined doses of colistin and flavomycin (0.5 + 16 mg kg^−1^, 2 + 16 mg kg^−1^, and 2 + 8 mg kg^−1^) via intraperitoneal injection after 1 h. Survival was monitored for 7 days. In the mouse thigh infection model, mice received a bacterial injection into their right thighs and were treated with either PBS, colistin (2 and 4 mg kg^−1^) , flavomycin (16 mg kg^−1^), or combinations of colistin and flavomycin (0.5 + 16 mg kg^−1^, 2 + 16 mg kg^−1^, and 4 + 16 mg kg^−1^) via intraperitoneal injection after 2 h. The mice were euthanized, and their right thighs were aseptically removed, homogenized, serially diluted, and plated on MH plates for viability assessment after 48 h.

### Statistics and reproducibility

Statistical analysis was performed using GraphPad Prism 8 software. All data were obtained from at least three independent experiments and presented as means ± SD, unless otherwise noted. In the *in vitro* studies, unpaired *t*-test between two groups or one-way ANOVA among multiple groups were used to calculate *P* values. In animal studies, significance of bacterial load in mouse thigh infection experiment and survival rates in mouse peritonitis–sepsis were analyzed by Mann–Whitney *U*-test or log-rank (Mantel–Cox) test, respectively.

## SUPPLEMENTAL MATERIAL

Supplemental Figures msystems.00109-24-s0001.pdf Figures S1 to S4.

Supplemental Tables msystems.00109-24-s0002.xlsx Tables S1-S4.

ASM does not own the copyrights to Supplemental Material that may be linked to, or accessed through, an article. The authors have granted ASM a non-exclusive, world-wide license to publish the Supplemental Material files. Please contact the corresponding author directly for reuse.

## References

[1] Laxminarayan R. 2014. Antibiotic effectiveness: balancing conservation against innovation. Science 345:1299–1301. doi:10.1126/science.1254163 25214620

[2] Markou N, Apostolakos H, Koumoudiou C, Athanasiou M, Koutsoukou A, Alamanos I, Gregorakos L. 2003. Intravenous colistin in the treatment of sepsis from multiresistant Gram-negative bacilli in critically ill patients. Crit Care 7:R78–R83. doi:10.1186/cc2358 12974973 PMC270720

[3] Falagas ME, Kasiakou SK, Saravolatz LD. 2005. Colistin: the revival of polymyxins for the management of multidrug-resistant Gram-negative bacterial infections. Clin Infect Dis 40:1333–1341. doi:10.1086/429323 15825037

[4] Liu Y-Y, Wang Y, Walsh TR, Yi L-X, Zhang R, Spencer J, Doi Y, Tian G, Dong B, Huang X, Yu L-F, Gu D, Ren H, Chen X, Lv L, He D, Zhou H, Liang Z, Liu J-H, Shen J. 2016. Emergence of plasmid-mediated colistin resistance mechanism mcr-1 in animals and human beings in China: a microbiological and molecular biological study. Lancet Infect Dis 16:161–168. doi:10.1016/S1473-3099(15)00424-7 26603172

[5] El-Sayed Ahmed MAE-G, Zhong L-L, Shen C, Yang Y, Doi Y, Tian G-B. 2020. Colistin and its role in the era of antibiotic resistance: an extended review (2000-2019). Emerg Microbes Infect 9:868–885. doi:10.1080/22221751.2020.1754133 32284036 PMC7241451

[6] Liu Y-Y, Zhou Q, He W, Lin Q, Yang J, Liu J-H. 2020. mcr-1 and plasmid prevalence in Escherichia coli from livestock. Lancet Infect Dis 20:1126. doi:10.1016/S1473-3099(20)30697-6 32979325

[7] Liu J-H, Liu Y-Y, Shen Y-B, Yang J, Walsh TR, Wang Y, Shen J. 2024. Plasmid-mediated colistin-resistance genes: mcr. Trends Microbiol 32:365–378. doi:10.1016/j.tim.2023.10.006 38008597

[8] Elbediwi M, Li Y, Paudyal N, Pan H, Li X, Xie S, Rajkovic A, Feng Y, Fang W, Rankin SC, Yue M. 2019. Global burden of colistin-resistant bacteria: mobilized colistin resistance genes study (1980–2018). Microorganisms 7:461. doi:10.3390/microorganisms7100461 31623244 PMC6843232

[9] Osei Sekyere J, Reta MA. 2020. Genomic and resistance epidemiology of Gram-negative bacteria in Africa: a systematic review and phylogenomic analyses from a one health perspective. mSystems 5:00897-20. doi:10.1128/mSystems.00897-20 PMC7687029 33234606

[10] Velkov T, Dai C, Ciccotosto GD, Cappai R, Hoyer D, Li J. 2018. Polymyxins for CNS infections: pharmacology and neurotoxicity. Pharmacol Ther 181:85–90. doi:10.1016/j.pharmthera.2017.07.012 28750947

[11] Tsuji BT, Landersdorfer CB, Lenhard JR, Cheah S-E, Thamlikitkul V, Rao GG, Holden PN, Forrest A, Bulitta JB, Nation RL, Li J. 2016. Paradoxical effect of polymyxin B: high drug exposure amplifies resistance in Acinetobacter baumannii. Antimicrob Agents Chemother 60:3913–3920. doi:10.1128/AAC.02831-15 27067330 PMC4914656

[12] Ni W, Shao X, Di X, Cui J, Wang R, Liu Y. 2015. In vitro synergy of polymyxins with other antibiotics for Acinetobacter baumannii: a systematic review and meta-analysis. Int J Antimicrob Agents 45:8–18. doi:10.1016/j.ijantimicag.2014.10.002 25465524

[13] MacNair CR, Stokes JM, Carfrae LA, Fiebig-Comyn AA, Coombes BK, Mulvey MR, Brown ED. 2018. Overcoming mcr-1 mediated colistin resistance with colistin in combination with other antibiotics. Nat Commun 9:458. doi:10.1038/s41467-018-02875-z 29386620 PMC5792607

[14] Zhang Q, Chen S, Liu X, Lin W, Zhu K. 2021. Equisetin restores colistin sensitivity against multi-drug resistant Gram-negative bacteria. Antibiotics (Basel) 10:10. doi:10.3390/antibiotics10101263 PMC8532683 34680843

[15] Graf FE, Palm M, Warringer J, Farewell A. 2019. Inhibiting conjugation as a tool in the fight against antibiotic resistance. Drug Dev Res 80:19–23. doi:10.1002/ddr.21457 30343487

[16] Kudo H, Usui M, Nagafuji W, Oka K, Takahashi M, Yamaguchi H, Tamura Y. 2019. Inhibition effect of flavophospholipol on conjugative transfer of the extended-spectrum β-lactamase and vanA genes. J Antibiot 72:79–85. doi:10.1038/s41429-018-0113-4 PMC6760635 30361635

[17] Poole TL, McReynolds JL, Edrington TS, Byrd JA, Callaway TR, Nisbet DJ. 2006. Effect of flavophospholipol on conjugation frequency between Escherichia coli donor and recipient pairs in vitro and in the chicken gastrointestinal tract. J Antimicrob Chemother 58:359–366. doi:10.1093/jac/dkl249 16847028

[18] Chen X, Wong C-H, Ma C. 2019. Targeting the bacterial transglycosylase: antibiotic development from a structural perspective. ACS Infect Dis 5:1493–1504. doi:10.1021/acsinfecdis.9b00118 31283163

[19] Ostash B, Walker S. 2010. Moenomycin family antibiotics: chemical synthesis, biosynthesis, and biological activity. Nat Prod Rep 27:1594–1617. doi:10.1039/c001461n 20730219 PMC2987538

[20] Clifton LA, Skoda MWA, Le Brun AP, Ciesielski F, Kuzmenko I, Holt SA, Lakey JH. 2015. Effect of divalent cation removal on the structure of gram-negative bacterial outer membrane models. Langmuir 31:404–412. doi:10.1021/la504407v 25489959 PMC4295546

[21] Fuse S, Tsukamoto H, Yuan Y, Wang T-S, Zhang Y, Bolla M, Walker S, Sliz P, Kahne D. 2010. Functional and structural analysis of a key region of the cell wall inhibitor moenomycin. ACS Chem Biol 5:701–711. doi:10.1021/cb100048q 20496948 PMC2912422

[22] Heaslet H, Shaw B, Mistry A, Miller AA. 2009. Characterization of the active site of S. aureus monofunctional glycosyltransferase (Mtg) by site-directed mutation and structural analysis of the protein complexed with moenomycin. J Struct Biol 167:129–135. doi:10.1016/j.jsb.2009.04.010 19416756

[23] Wang X, He J, Le K. 2018. Making point mutations in Escherichia coli BL21 genome using the CRISPR-Cas9 system. FEMS Microbiol Lett 365:14. doi:10.1093/femsle/fny060 29596631

[24] Zhong Z, Zhou S, Liang Y, Wei Y, Li Y, Long T, He Q, Li M, Zhou Y, Yu Y, Fang L, Liao X, Kreiswirth BN, Chen L, Ren H, Liu Y, Sun J. 2023. Natural flavonoids disrupt bacterial iron homeostasis to potentiate colistin efficacy. Sci Adv 9:23. doi:10.1126/sciadv.adg4205 PMC10256158 37294761

[25] Brennan-Krohn T, Pironti A, Kirby JE. 2018. Synergistic activity of colistin-containing combinations against colistin-resistant Enterobacteriaceae. Antimicrob Agents Chemother 62:e00873-18. doi:10.1128/AAC.00873-18 30061285 PMC6153801

[26] Stokes JM, MacNair CR, Ilyas B, French S, Côté J-P, Bouwman C, Farha MA, Sieron AO, Whitfield C, Coombes BK, Brown ED. 2017. Pentamidine sensitizes Gram-negative pathogens to antibiotics and overcomes acquired colistin resistance. Nat Microbiol 2:17028. doi:10.1038/nmicrobiol.2017.28 28263303 PMC5360458

[27] Zhu X-Q, Liu Y-Y, Wu R, Xun H, Sun J, Li J, Feng Y, Liu J-H. 2021. Impact of mcr-1 on the development of high level colistin resistance in Klebsiella pneumoniae and Escherichia coli. Front Microbiol 12:666782. doi:10.3389/fmicb.2021.666782 33981294 PMC8108134

[28] Wu R, Yi L, Yu L, Wang J, Liu Y, Chen X, Lv L, Yang J, Liu J-H. 2018. Fitness advantage of mcr-1–bearing IncI2 and IncX4 plasmids in vitro. Front Microbiol 9:331. doi:10.3389/fmicb.2018.00331 29535696 PMC5835064

[29] Yang J, Wang H-H, Lu Y, Yi L-X, Deng Y, Lv L, Burrus V, Liu J-H. 2021. A ProQ/FinO family protein involved in plasmid copy number control favours fitness of bacteria carrying mcr-1-bearing IncI2 plasmids. Nucleic Acids Res 49:3981–3996. doi:10.1093/nar/gkab149 33721023 PMC8053102

[30] Humphries R, Bobenchik AM, Hindler JA, Schuetz AN. 2021. Overview of changes to the clinical and laboratory standards institute performance standards for antimicrobial susceptibility testing, M100. J Clin Microbiol 59:e0021321. doi:10.1128/JCM.00213-21 34550809 PMC8601225

[31] Odds FC. 2003. Synergy, antagonism, and what the chequerboard puts between them. J Antimicrob Chemother 52:1. doi:10.1093/jac/dkg301 12805255

