# Supplementary material for: Flavomycin restores colistin susceptibility in multidrug-resistant Gram-negative bacteria
Source: mSystems. 2024 May 2;9(6):e00109-24. doi: 10.1128/msystems.00109-24 (PMC11237640; doi:10.1128/msystems.00109-24)
Supplement: Supplemental Figures — Figures S1 to S4. [file msystems.00109-24-s0001.pdf]

Supplementary figures

Fig S1: Impacts of flavomycin on conjugation frequencies. Flavomycin exhibits a dose-dependent inhibition of conjugation frequencies in most E. coli strains. Bars are painted in various colors according to different resistant genes: mcr-1-positive (orange), blaNDM-positive (blue), blaCTX-M-positive (gray) and mcr-1 & blaCTX-M positive (green). Data points are from three independent experiments, each done in duplicate.

Fig S2: Influence of Mg2+ on the synergy and impacts of mutations on bacterial growth. a: The synergy of flavomycin and colistin still existed when low Mg2+ concentration was added exogenously, but the synergy was relatively weakened compared to the group without Mg2+. However, when exogenous Mg2+ was added at a concentration greater than 15mM, the synergy of flavomycin and colistin disappeared. b: Impacts of mutations on bacterial growth. The growth of bacteria with mutations were not affected.

Fig S3: Strategy of CRISPR/Cas9-mediated genome editing in E. coli. a An overview of the successive two-sgRNA strategy for genome editing with CRISPR-Cas9 in E. coli. A two-step genome editing strategy was utilized to specifically introduce a single amino acid mutation (E233Q) in the mcrB gene of BW25113 strain. To generate the Cas9/sgRNA vectors for targeted nucleotide point mutation, a 1 kb fragment encompassing the coding region, with the Cas9/sgRNA cleavage site positioned in the middle, was PCR amplified using primers listed in Table S4. Two sgRNAs were designed to target the region adjacent to the desired amino acid sites (E233) (Table S4). Synthetic oligonucleotides and PCR amplification were employed to introduce five nucleotide mutations in the sgRNA-binding site of the donor template, while keeping the amino acids unchanged. This was done to prevent repeated binding of sgRNA to the target site within the donor template. The mutant strains (E233Q) were confirmed with Sanger (DNA) sequencing. All plasmids used in this study are available from the authors upon request. b The scheme and sanger sequencing confirmation of point mutation. Sequencing shows successful introduction of a point mutation in PBP1b. WT, wild-type.

Fig S4: The intracellular accumulation of flavomycin among different strains. a: The intracellular accumulation of flavomycin was higher when it was treated with flavomycin in combination with colistin, PMBN, or EDTA, which can increase the OM permeability. The percentage increase in flavomycin accumulation was determined by dividing the difference in intracellular concentration between combined therapy and monotherapy by the intracellular concentration of mono-therapy. The results indicate that the intracellular accumulation of flavomycin in E. coli can be increased when flavomycin is combined with drugs that increase the OM permeability. However, whether treated with flavomycin alone (FLA16, b) or in combination with other drugs (FLA16 + CL1, FLA16 + CL2, FLA16 + PMBN100, and FLA16 + PMBN60, c-f), the knockout strain (BW25113ΔwaaC) had an increased intracellular accumulation of flavomycin compared to the wild-type strain under the same treatment, about 11.5%~85.4%. In contrast, the mutant strains (BW25113 (E233Q) or BW25113ΔwaaC (E233Q)) had a decreased intracellular accumulation of flavomycin compared to the wild-type strain under the same treatment.

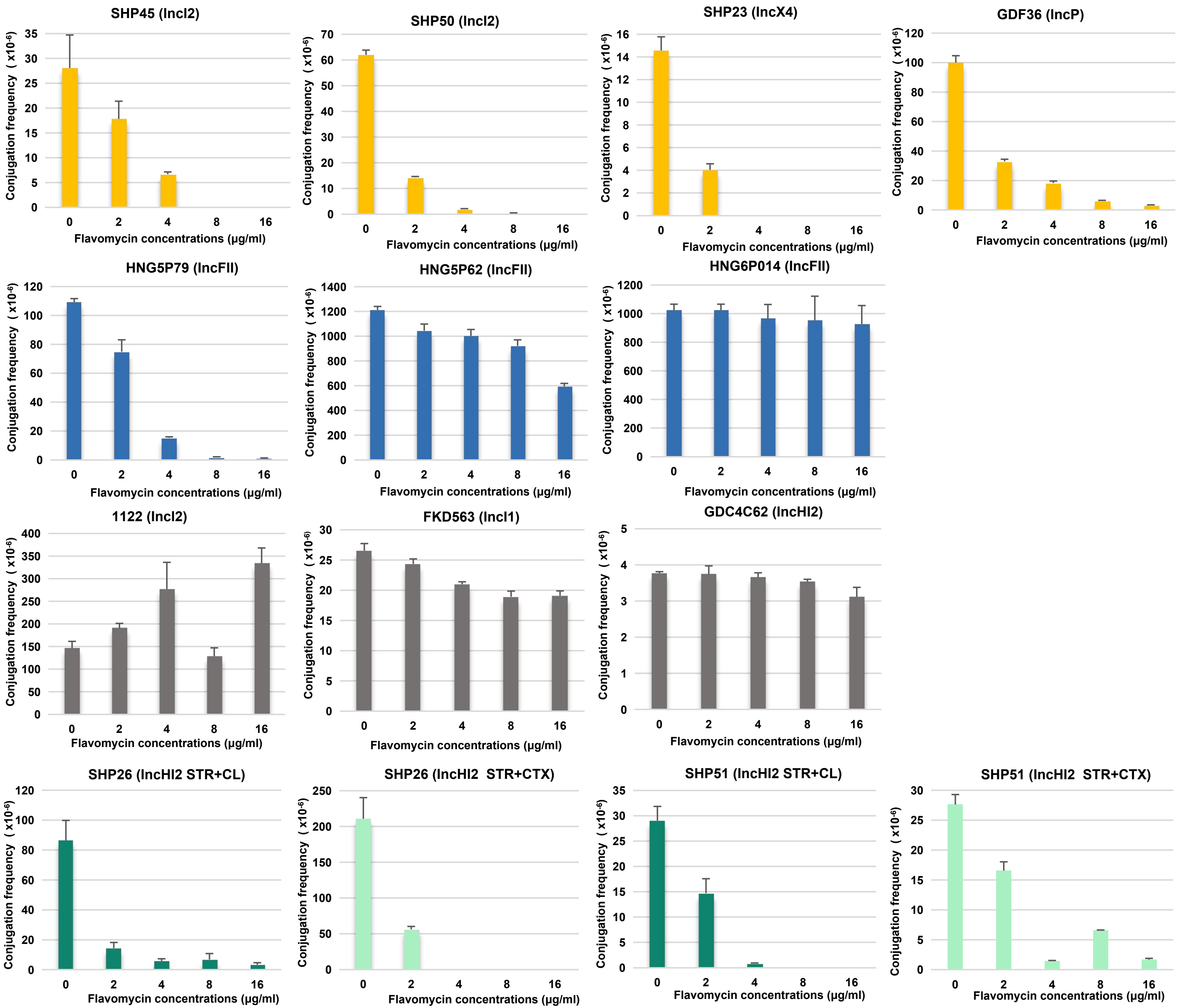
SHP45 (IncI2)
SHP50 (IncI2)
SHP23 (IncX4)
GDF36 (IncP)
HNG5P79 (IncFII)
HNG5P62 (IncFII)
HNG6P014 (IncFII)
1122 (IncI2)
FKD563 (IncI1)
GDC4C62 (IncHI2)
SHP26 (IncHI2 STR+CL)
SHP26 (IncHI2 STR+CTX)
SHP51 (IncHI2 STR+CL)
SHP51 (IncHI2 STR+CTX)
Conjugation frequency (x10⁻⁶)
Flavomycin concentrations (μg/ml)
0
2
4
8
16

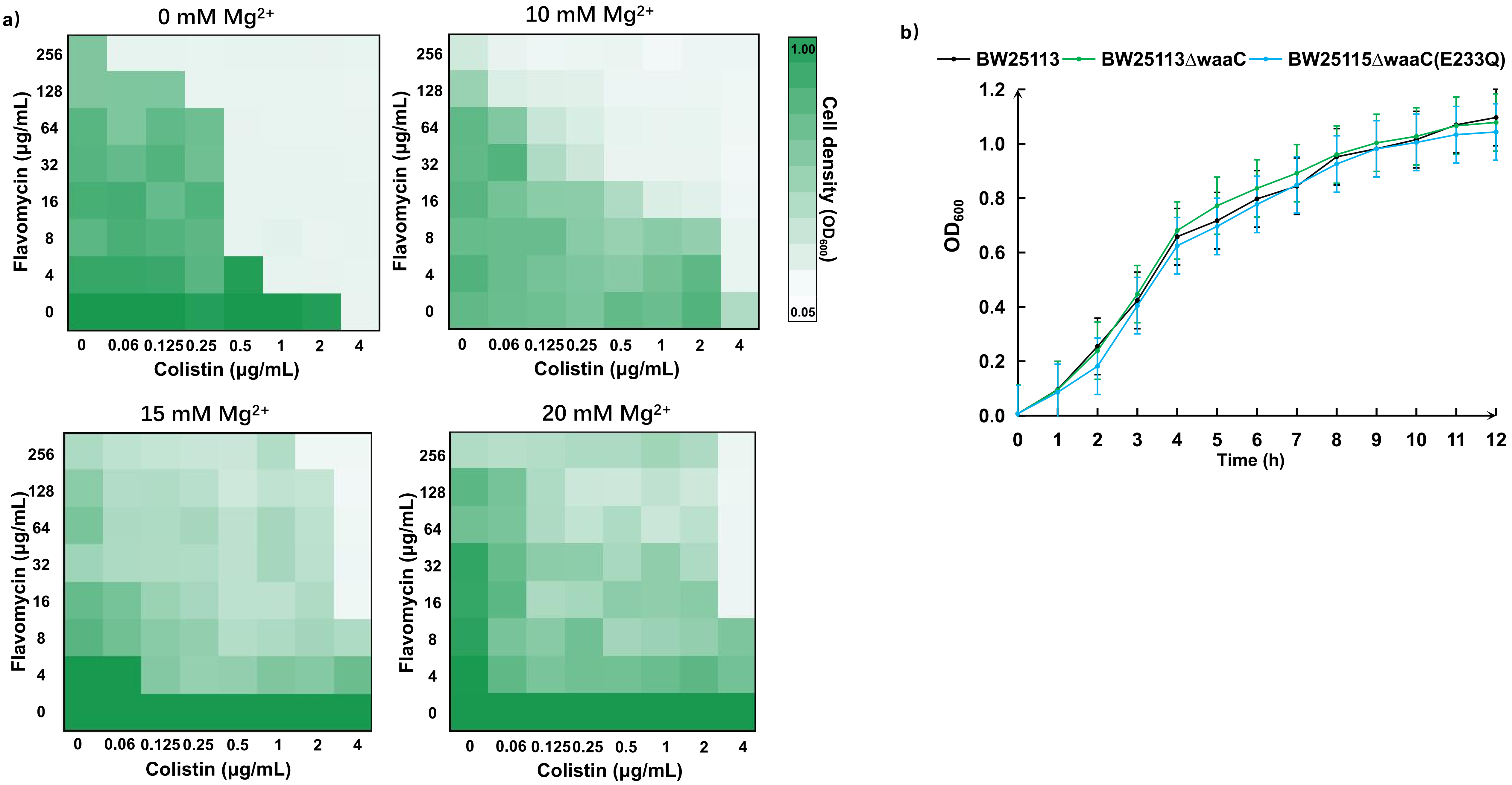

a)
0 mM Mg2+
10 mM Mg2+
15 mM Mg2+
20 mM Mg2+
Flavomycin (μg/mL)
256
128
64
32
16
8
4
0
Colistin (μg/mL)
0
0.06
0.125
0.25
0.5
1
2
4
Cell density (OD600)
1.00
0.05
b)
BW25113
BW25113ΔwaaC
BW25115ΔwaaC(E233Q)
OD600
1.2
1.0
0.8
0.6
0.4
0.2
0.0
Time (h)
0
1
2
3
4
5
6
7
8
9
10
11
12

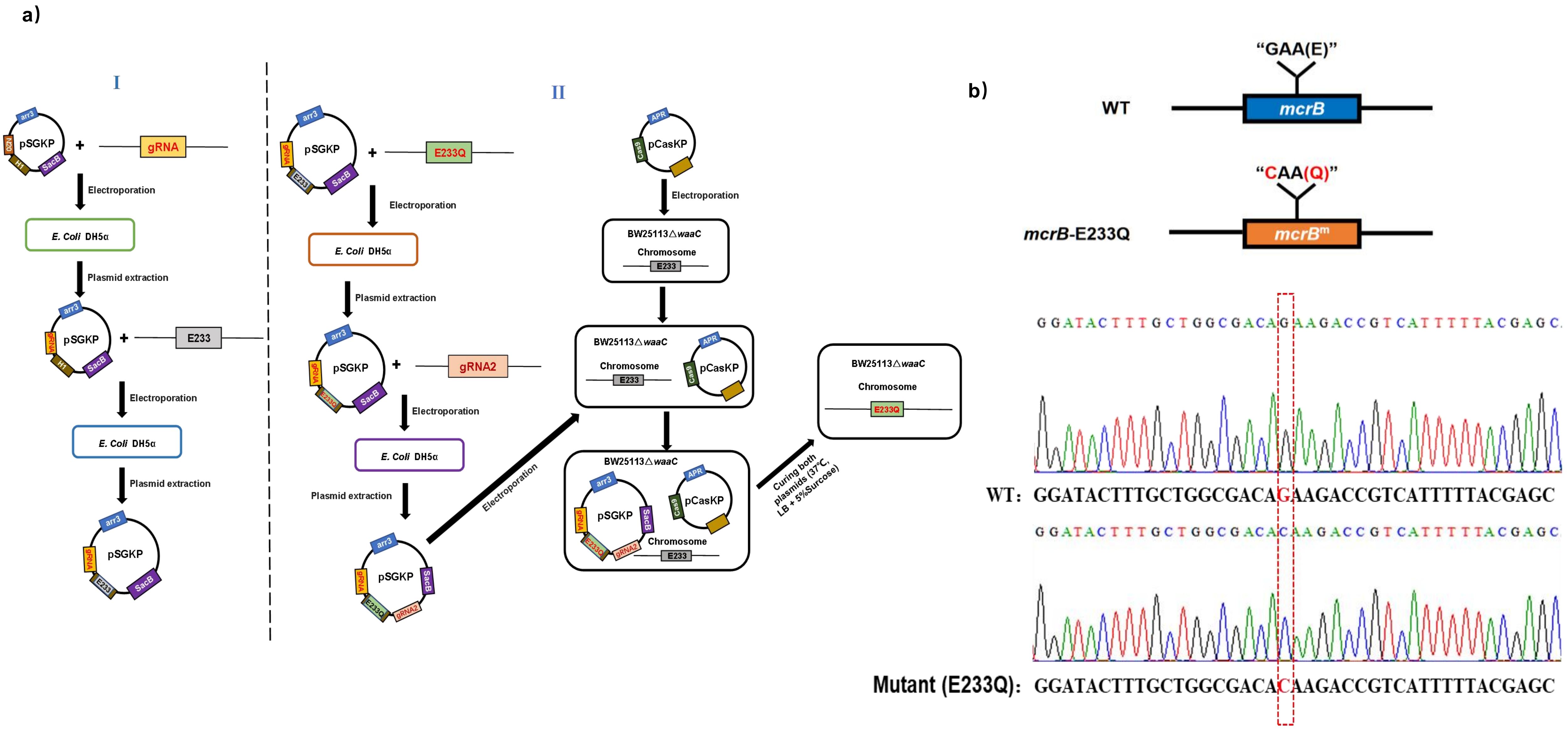
a)
I
II
pSGKP
arr3
N20
H1
SacB
gRNA
Electroporation
E. Coli DH5α
Plasmid extraction
E233
E233Q
gRNA2
pCasKP
APR
Cas9
BW25113△waaC
Chromosome
Curing both plasmids (37°C, LB + 5%Surcose)
b)
"GAA(E)"
WT
mcrB
"CAA(Q)"
mcrB-E233Q
mcrBm
WT: GGATACTTTGCTGGCGACAGAAGACCGTCATTTTTACGAGC
Mutant (E233Q): GGATACTTTGCTGGCGACACAAGACCGTCATTTTTACGAGC

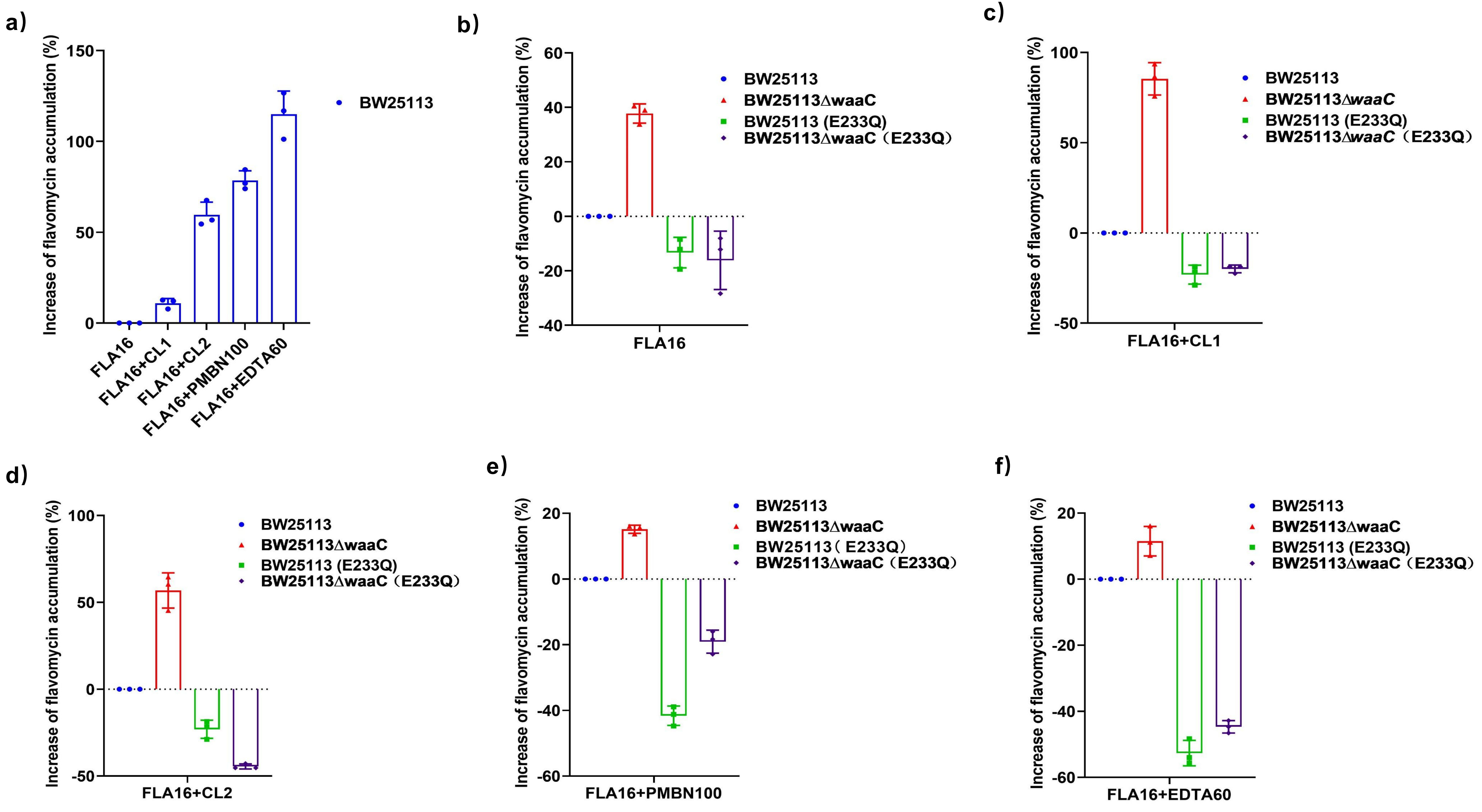
a)
Increase of flavomycin accumulation (%)
150
100
50
0
BW25113
FLA16
FLA16+CL1
FLA16+CL2
FLA16+PMBN100
FLA16+EDTA60
b)
Increase of flavomycin accumulation (%)
60
40
20
0
-20
-40
BW25113
BW25113ΔwaaC
BW25113 (E233Q)
BW25113ΔwaaC（E233Q）
FLA16
c)
Increase of flavomycin accumulation (%)
100
50
0
-50
BW25113
BW25113ΔwaaC
BW25113 (E233Q)
BW25113ΔwaaC（E233Q）
FLA16+CL1
d)
Increase of flavomycin accumulation (%)
100
50
0
-50
BW25113
BW25113ΔwaaC
BW25113 (E233Q)
BW25113ΔwaaC（E233Q）
FLA16+CL2
e)
Increase of flavomycin accumulation (%)
20
0
-20
-40
-60
BW25113
BW25113ΔwaaC
BW25113（ E233Q）
BW25113ΔwaaC（E233Q）
FLA16+PMBN100
f)
Increase of flavomycin accumulation (%)
20
0
-20
-40
-60
BW25113
BW25113ΔwaaC
BW25113 (E233Q)
BW25113ΔwaaC（E233Q）
FLA16+EDTA60
